# Multidrug-Resistant Lineage of Enterotoxigenic *Escherichia coli* ST182 With Serotype O169:H41 in Airline Waste

**DOI:** 10.3389/fmicb.2021.731050

**Published:** 2021-09-07

**Authors:** Veronica M. Jarocki, Stefanie Heß, Kay Anantanawat, Thomas U. Berendonk, Steven P. Djordjevic

**Affiliations:** ^1^iThree Institute, University of Technology Sydney, Sydney, NSW, Australia; ^2^Institute of Microbiology, Technische Universität Dresden, Dresden, Germany; ^3^Institute of Hydrobiology, Technische Universität Dresden, Dresden, Germany

**Keywords:** Enterotoxigenic *Escherichia coli* (ETEC), ST182, O169:H41, airplane waste, antimicrobial resistance (AMR), *bla*_CTX–M_, traveller’s diarrhoea

## Abstract

Enterotoxigenic *Escherichia coli* (ETEC) is the primary aetiologic agent of traveller’s diarrhoea and a significant cause of diarrhoeal disease and death in developing countries. ETEC O169:H41 strains are known to cause both traveller’s diarrhoea and foodborne outbreaks in developed countries and are cause for concern. Here, whole-genome sequencing (WGS) was used to assemble 46 O169:H41 (ST182) *E. coli* draft genomes derived from two airplane waste samples sourced from a German international airport. The ST182 genomes were compared with all 84 publicly available, geographically diverse ST182 genomes to construct a core genome-based phylogenetic tree. ST182 isolates were all phylogroup E, the majority serotype O169:H41 (*n* = 121, 93%) and formed five major clades. The airplane waste isolates differed by an average of 15 core SNPs (range 0–45) but their accessory genome content was diverse. While uncommon in other ST182 genomes, all airplane-derived ST182 isolates carried: (i) extended-spectrum β-lactamase gene *bla*_CTX–M–__15_ notably lacking the typical adjacent IS*Ecp1*; (ii) *qnrS1* and the S83L mutation in *gyrA*, both conferring resistance to fluoroquinolones; and (iii) a class 1 integron structure (IS*26*-*intI1*_Δ__648_-*dfrA17*-*aadA5*-*qacE*Δ*1*-*sul1*-ORF-*srpC*-*padR*-IS*6100*-*mphR*-*mrx*-*mphA*-IS*26*) identified previously in major extraintestinal pathogenic *E. coli* STs but not in ETEC. ST182 isolates carried ETEC-specific virulence factors STp + CS6. Adhesin/invasin *tia* was identified in 89% of aircraft ST182 isolates (vs 23%) and was located on a putative genomic island within a hotspot region for various insertions including PAI I_536_ and plasmid-associated transposons. The most common plasmid replicons in this collection were IncFII (100%; F2:A-:B-) and IncB/O/K/Z (89%). Our data suggest that potentially through travel, *E. coli* ST182 are evolving a multidrug-resistant profile through the acquisition of class 1 integrons and different plasmids.

## Introduction

Throughout history, travel has played a key role in disseminating infectious diseases and with air travel now servicing over four billion passengers annually (ICAO, 2018), travellers in unprecedented numbers and speeds continue to shape the emergence and spread of disease to ever broader geographic locations (Institute of Medicine Forum on Microbial Threats, 2010). More recently, international travel has also been shown to contribute to the rise of antimicrobial resistance (AMR), particularly through the acquisition of extended-spectrum β-lactamase (ESBL)-producing *Enterobacteriaceae* (Hassing et al., 2015; Woerther et al., 2017). Indeed, the rates of ESBL-producing *Enterobacteriaceae* acquisition during travel are alarming, with a recent study reporting up to 75 and 44% acquisition in travellers returning from Asia and Africa respectively (Arcilla et al., 2017). The same study found a strong correlation between ESBL-producing *Enterobacteriaceae* acquisition, diarrhoea during travel, and antimicrobial use, particularly fluoroquinolones. Similarly, another study found that 80% of travellers returning from southern Asia that used antimicrobials to treat diarrhoea whilst abroad had subsequently acquired ESBL-producing *Enterobacteriaceae*, as opposed to 47% of travellers that experienced diarrhoea without antimicrobial treatment, and 23% of travellers that reported neither diarrhoea nor antimicrobial use (Kantele et al., 2015). Another recent study involving real-time colonisation dynamics indicated that 70% of participant travellers returning from southeast Asia were colonised with ESBL-producing *Enterobacteriaceae* but that all had acquired ESBL-producing *Enterobacteriaceae* at one point in their travels (Kantele et al., 2021). Perhaps unsurprisingly, airplane sewage has been found to be an extraordinary source of antimicrobial resistance genes (ARGs), including ESBL genes, such as *bla*_CTX–M_ (Heß et al., 2019).

Traveller’s diarrhoea is the most common health problem afflicting visitors to lower socioeconomic regions, affecting up to 40% of travellers (Leung et al., 2019). While most cases are mild and self-limiting, typically resolving within two days (Tribble, 2017), severe cases can present with cholera-like watery stools, result in the loss of 10 L of fluids daily, and can lead to hospitalisation and even death (Margulieux et al., 2018; Mirhoseini et al., 2018; Hosangadi et al., 2019). Antimicrobial treatment for traveller’s diarrhoea reduces both symptoms and duration of the illness (Diemert, 2006). Thus, fluoroquinolones, macrolides and bacterial RNA polymerase-binding rifaximin are antimicrobials commonly prescribed prior to departure for travellers to carry and use in the event of moderate to severe illness (Taylor et al., 2017; Schweitzer et al., 2019). While the provision of an empiric self-treatment option can be appealing to travellers hoping to avoid travel plan disruptions and hospitalisations in developing countries, the practice can lead to inappropriate usage in mild cases (Vilkman et al., 2019) and as previously noted, antimicrobial treatment of traveller’s diarrhoea increases the risk of acquiring ESBL-producing *Enterobacteriaceae*.

The leading aetiological agent of traveller’s diarrhoea is enterotoxigenic *Escherichia coli* (ETEC), accounting for approximately 60% of all cases (Mirhoseini et al., 2018). However, ETEC does not only afflict travellers, but is a leading cause of diarrhoeal disease in developing countries and causes significantly more deaths than cholera and typhoid fever (Lamberti et al., 2014; Hosangadi et al., 2019). In humans, ETEC are distinguished from other *E. coli* pathotypes by the production of heat-labile enterotoxins (LT; LTIh, LTIIa, LTIIb, LTIIc variants) and/or heat-stable enterotoxins (STa; STp, STh variants), and colonisation factors (CFs), which are also known as coli surface (CS) antigens (Wang et al., 2019). There are currently over 29 different CFs identified in ETEC strains with more believed yet to be characterised (Nada et al., 2011; Ban et al., 2015; Zeinalzadeh et al., 2017), however, CFA/I and CS1-CS6 are the most prevalent (Cho et al., 2014). Apart from some LT variants, which are carried on prophages, all CFs and other enterotoxins are carried on plasmids (F-:A-:B-) in various combinations (Regua-Mangia et al., 2004; Wang et al., 2019).

While ETEC infections are predominately associated with visitors to, and residents of, developing countries, some ETEC serotypes have been linked to food-borne outbreaks in developed countries. One such serotype is O169:H41 (sequence type [ST] 182), which was first described causing an outbreak in 1991 in Japan and 15 subsequent outbreaks in Japan between 1991 and 1994 (Nishikawa, 1995; Nishikawa et al., 1998). In the United States, of 16 documented ETEC outbreaks between 1996 and 2003, O169:H41 was identified in ten, and was solely responsible for six (Beatty et al., 2004). In 2006, another O169:H41 outbreak occurred in the United States affecting 36 people and was linked to contaminated coleslaw at a catered event (Devasia et al., 2006). A more recent O169:H41 outbreak in Japan affected 102 people during a 2 day festival in 2012 (Harada et al., 2013) and in the same year, this serotype caused an outbreak linked to contaminated kimchi provided at seven schools in Korea, affecting 1642 people (Cho et al., 2014).

Despite the strong association with gastroenteritis outbreaks, to date no genomic comparisons of O169:H41/ST182 strains, nor reports on potential AMR gene carriage have been published. Here we used whole genome sequencing (WGS) to characterise 46 ST182 isolates collected from the sewage of aircraft landing at a large German international airport in 2016. We performed phylogenetic and pangenomic comparative analyses with other publicly available ST182 genomes and screened for the presence of virulence-associated genes (VAGs), antimicrobial resistance genes (ARGs), chromosomal mutations conferring AMR, and plasmid replicons. Furthermore, AMR regions and an invasin-rich genomic island present in airplane-derived isolates were characterised.

## Materials and Methods

### Sample Collection and DNA Extraction

Airplane-borne sewage was sampled at two locations on a single day in 2016: one sample was gathered from a vacuum truck which collected waste from aircraft arriving from Mombasa, Canada, and Singapore. The second sample was taken from sewerage where the contents of aircraft tanks was piped to a sewage treatment plant. Thus, the second sample comprised waste from multiple airplanes landing at an international airport in Germany. The two samples were stored in 1 L sterile glass bottles at 4°C and processed within 24 h. To isolate *E. coli*, suitable dilutions were plated on mFC agar (Carl Roth, Karlsruhe, Germany). After 18 ± 2 h of incubation at 44°C, blue colonies were streaked on Brilliance agar (Oxoid, Wesel, Germany) and grown overnight at 37°C to obtain pure cultures. To identify the isolates as *E. coli*, colony PCR was performed as described previously to amplify a species-specific fragment of the *yccT* gene (Clifford et al., 2012; Heß et al., 2018). The isolates were subsequently grown overnight in LB-broth (Carl Roth, Karlsruhe, Germany) and the DNA was isolated using the DNeasy 96 Blood and Tissue Kit (Qiagen, Hilden, Germany) following manufacturer instructions.

### Phenotypic Resistance Testing

The susceptibility of the *E. coli* isolates was tested against 24 antibiotics commonly used to treat *E. coli* infections (namely, ampicillin, amoxicillin-clavulanic acid, piperacillin, ticarcillin, cefepime, cefuroxime, cefoxitin, cefpodoxime, ceftazidime, cefuroxime, doripenem, ertapenem, imipenem, meropenem, ciprofloxacin, levofloxacin, norfloxacin, amikacin, gentamicin, netilmicin, tobramycin, tigecycline, chloramphenicol, sulfamethoxazole-trimethoprim). The agar diffusion tests followed the EUCAST v6.0 guidelines (eucast.org/) using *E. coli* ATCC 25922 as a quality control. Applying the clinical breakpoints defined by EUCAST, the isolates were classified as resistant, intermediate, or susceptible.

### Whole-Genome Sequencing

Library preparation was done by the iThree Core Sequencing facility, University of Technology Sydney, following the adapted Nextera Flex library preparation kit process, Hackflex (Gaio et al., 2019). Briefly, genomic DNA was quantitatively assessed using Quant-iT picogreen dsDNA assay kit (Invitrogen, United States). The sample was normalised to the concentration of 1 ng/μl. 10 ng of DNA was used for library preparation. After tagmentation, the tagmented DNA was amplified using the facility’s custom designed i7 and i5 barcodes, with 12 cycles of PCR.

Due to the number of samples, the quality control for the samples was done by sequencing a pool of samples using MiSeq V2 nano kit – 300 cycles. Briefly, after library amplification, 3 μl of each library was pooled into a library pool. The pool was then cleaned up using SPRIselect beads (Beckman Coulter, United States). The pool was sequenced using MiSeq V2 nano kit (Illumina, United States). Based on the sequencing data generated, the read count for each sample was used to identify any failed libraries (<100 reads) and normalised sample amounts to ensure equal representation in the final pool. The final pool was sequenced on one lane of Illumina Novaseq S4 flow cell, 2 × 150 bp at the Ramaciotti Centre for Genomics (University of New South Wales, Australia).

The resulting *E. coli* draft genomes were assembled using Shovill v1.0.4 (github.com/tseemann/shovill) with default settings and trimming option and underwent quality control (QC) using assembly-stats (github.com/sanger-pathogens/assembly-stats).

### Genome Annotation

Automated annotations were generated using Prokka v1.14.6 (Seemann, 2014) and managed via SnapGene v4.1.9 (snapgene.com). Genomic islands (GIs) were identified using IslandViewer 4 (Bertelli et al., 2017) using *E. coli* ST182 strain 2014EL-1345-2 as the reference genome, and phage elements were identified using the Phage Search Tool (PHAST) (Zhou et al., 2011).

### Phylogenetic Analysis

Additional publicly deposited *E. coli* ST182 genomes were sourced from the Enterobase database (Zhou et al., 2019) (*n* = 87; extracted 02/05/20) and Sequence Read Archive (SRA) FASTQ files were downloaded using parallel-fastq-dump (github.com/rvalieris/parallel-fastq-dump). Reads were then assembled using Shovill. *E. coli* ST182 derived from Enterobase are referred to throughout the manuscript as “strains,” while from this collection they are referred to as “isolates” of a novel ST182 lineage.

The *E. coli* ST182 pangenome was calculated using Panaroo (Tonkin-Hill et al., 2020) in strict mode after a pre-processing QC step using a Panaroo packaged Mash wrapper script. Any genome outliers with comparatively unusual number of contigs or genes (i.e., number of genes >5100 or <4400; and number of contigs >300) were removed from all downstream analyses. A core gene alignment (4,123,297 bp in length) was also generated using Panaroo (default settings) and used to build a maximum-likelihood phylogenetic tree of all *E. coli* ST182 sequences via IQ-TREE 2 (Minh et al., 2020) in which ModelFinderPlus (-m MFP) was used to determine the best-fit model and single branch test performed using ultrafast bootstrap (1000 iterations). The pangenome was visualised using Phandango v1.3.0 (Hadfield et al., 2018) and the gene presence/absence matrix generated by Panaroo was used in Scoary (Brynildsrud et al., 2016) for pangenome-wide association studies.

Single nucleotide polymorphism (SNP)-based phylogenetic analyses were performed using Parnsp v1.1.2 (Treangen et al., 2014) with recombination filtering (-x). *E. coli* ST182 SS_2_A12 (GCA_015200705.1) was used as the reference to produce an alignment (85% of total length; 4,325,083 bp) to airplane-only isolates and the completed genome of ST182 strain F6326-C1 (GCA_002741495.1) was the reference used to produce an alignment to all ST182 sequences. Snp-dists v0.7.0 was then used on both alignments to calculate pairwise SNP distances. All trees were visualised using the Interactive Tree of Life (iTOL v4) (Letunic and Bork, 2019).

### Genotyping, Serotyping and Phylogenetic Classification

STs, serogroups and phylogroups for all genomes analysed in this study were determined *in silico* using Achtman 7 multilocus sequencing typing (MLST) v2.0 (Larsen et al., 2012), SerotypeFinder v2.0 (Joensen et al., 2015), and Clermont Typing (Clermont et al., 2013), respectively. Typing of *fimH* alleles was performed using FimTyper v1.0 (Roer et al., 2017). ABRicate (github.com/tseemann/abricate) was used to screen for VAGs, ARGs, chromosomal point mutations conferring AMR, and plasmid replicons using the following reference databases: Virulence Factor DataBase (VFDB) (Liu et al., 2019), ResFinder v4.1 (Zankari et al., 2012), the Comprehensive Antibiotic Resistance Database (CARD) (Alcock et al., 2020), PointFinder v4.1 (Zankari et al., 2017) and PlasmidFinder v2.1 (Carattoli and Hasman, 2020). An additional custom database with AMR-associated insertion sequences (IS), class 1, 2 and 3 integrases, and additional extraintestinal pathogenic *E. coli* (ExPEC)-associated VAGs was also utilised and can be accessed at github.com/CJREID/custom_DBs. ABRicate was executed using default parameters (minimal coverage ≥ 80%; minimal identity ≥ 80%), except when screening for truncated *intI1* genes where minimal coverage was lowered to > 60%.

The *tia-*containing region was screened for in *E. coli* ST182 genomes using the nucleic sequence of this region derived from isolate SS_1_H2 contig 15 (start: 72, 824, end: 133, 647). This sequence was also used to search the NCBI database using BLASTn. A comparison of identified *tia*-containing regions was made using EasyFig (Sullivan et al., 2011). BLASTn was also used to determine whether AMR-regions characterised in this collection were present in other genomes deposited into NCBI. Associated metadata for 100% identity hits was pulled from Genbank (Leray et al., 2019) and PLSDB v2020_03_04 (Galata et al., 2019).

To infer *E. coli* ST182 strain 2014EL-1345-2 plasmid carriage in this collection, short-reads from each isolate were mapped to plasmid unnamed 1 (NZ_CP024224.1), unnamed 2 (NZ_CP024225.1), unnamed 3 (NZ_CP024226.1) and unnamed 4 (NZ_CP024227.1) using Burrows-Wheeler (BWA) v0.7.17 (Li and Durbin, 2009) and converted to BAM files using SAMtools v01.1.18 (Li et al., 2009). A bespoke script, available at github.com/maxlcummins/APEC-MGEN-2018, was used to produce a histogram of read-depth as a function of reference coordinate and clustered based on their Euclidean distances, and then used to generate a heatmap.

### Statistical Analysis

A pairwise genome distance matrix was generated using Mash (Ondov et al., 2016) and used to create a classical (metric) multidimensional scaling (MDS) plot using R Studio v4.0.2 and the gglot2 v3.3.0 package (ggplot2.tidyverse.org/). MDS plots for VAGs and ARGs were also created in R Studio, using standard R package functions dist and cmdscale in conjunction with gene presence/absence matrices (1 = present; 0 = absent).

## Results

### Genome Selection, Assembly, and QC

A total of 71 isolates (13 ST lineages) from the two airplane waste samples were sequenced. ST182 made up 65% (*n* = 46) of all the isolates and are the subject of this study. The 46 ST182 genomes ranged in size from 4,988,334 bp to 5,327,796 bp, with a mean size of 5,208,154 bp. The number of contigs per genome ranged from 145 to 254, with a mean of 183. Read depth ranged from 58× to 124×, with a mean of 85×. Full assembly statistics can be viewed in Supplementary Data 1.

### *Escherichia coli* ST182 Phylogeny

**FIGURE 1:**
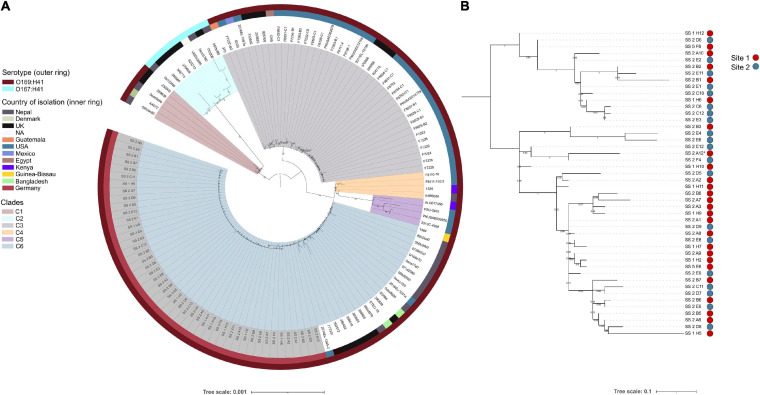
*E. coli* ST182 phylogeny. **(A)** Mid-point root maximum-likelihood tree by core genome alignment showing genetic relatedness between 130 ST182 genomes. Isolates from this collection have tip label shaded in gray. Tree generated using IQTree2. **(B)** Mid-point rooted SNP-based phylogeny tree showing topology of the 46 airplane waste isolates only. Site 1: vacuum truck with waste from aircraft arriving from Mombasa, Canada, and Singapore. Site 2: Sewage from mixed aircraft tanks. Tree generated using Parsnp. Both trees visualised using iTOL.

To ascertain the genetic relatedness of the airplane waste isolates to other *E. coli* ST182 strains all publicly available ST182 genomes (*n* = 84; metadata available in Supplementary Data 2) were used to construct a core genome-based phylogenetic tree using a core gene alignment 4,123,297 bp in length (Figure 1A). All airplane waste isolates were serotype O169:H41, as were the majority of ST182 strains (*n* = 121, 93%). These formed five major clades (Figure 1A) – however, a branch comprising of nine O167:H41 strains, formed its own clade (clade 2/C2). Airplane waste isolates were all situated in clade 6, with the closest relative outside the collection was 2014EL-1345-2 a human-sourced ETEC from the United States collected in 2014. All *E. coli* ST182 were typed as phylogroup E. Regarding *fimH* alleles, 58% (*n* = 75) carried *fimH*30, 31% (*n* = 40) carried *fimH*54, and 12% (*n* = 15) had no identified *fimH* gene. All airplane waste ST182 isolates carried *fimH*30. From available metadata, *E. coli* ST182 were most frequently isolated in Germany (*n* = 46, 35%; this collection), the United States (*n* = 38, 29%), the United Kingdom (*n* = 20, 15%) and Nepal (*n* = 16, 12%), and most originated from human samples (69%), however, one strain came from lettuce (strain PSU-0403; United States) and one from poultry (strain ALQ017456; Kenya), both of which reside in clade 5.

To view airplane waste isolate topology, a SNP-based phylogeny was also constructed (Figure 1B) using 85% (4325083 bp) of the reference genome SS_2_A12 to identify SNP sites. Despite being sampled from two different sites, these isolates differed by an average of 15 core SNPs (range 0–45) (Pairwise SNP distance matrix in Supplementary Data 3). A SNP analysis across all ST182 genomes was performed using 84% (4332006 bp) of the oldest completed genome as reference (strain F6326-C1; collected 1998; clade 3). With the exception of clade 5 isolates, SNP distances within clades were generally low, ranging from on average 51 SNPs for clade 6 to 111 SNPs for clade 2 (Table 1), with the exception of clade 5 isolates which differed by an average of 302 SNPs. SNP distances between clades were much higher, the greatest distance being between clade 2 and clade 5 isolates at an average of 6300 SNPs (Table 1).

**TABLE 1:** Average SNP distances across *E. coli* ST182 clades (Range in brackets).

	Clade 1	Clade 2	Clade 3	Clade 4	Clade 5	Clade 6
**Clade 1**	65 (20–95)					
**Clade 2**	3410 (3325–3469)	111 (7–199)				
**Clade 3**	2088 (2028–2200)	5417 (5331–5535)	86 (0–235)			
**Clade 4**	2486 (2418–2540)	5815 (5721–5875)	1815 (1747–1926)	77 (8–108)		
**Clade 5**	2980 (2922–3019)	6300 (6215–6344)	2309 (2247–2405)	597 (534–637)	302 (1–462)	
**Clade 6**	2562 (2459–2618)	5893 (5762–5948)	1895 (1788–2004)	169 (59–223)	680 (575–717)	51 (0–202)

### *Escherichia coli* ST182 Pangenome

**FIGURE 2:**
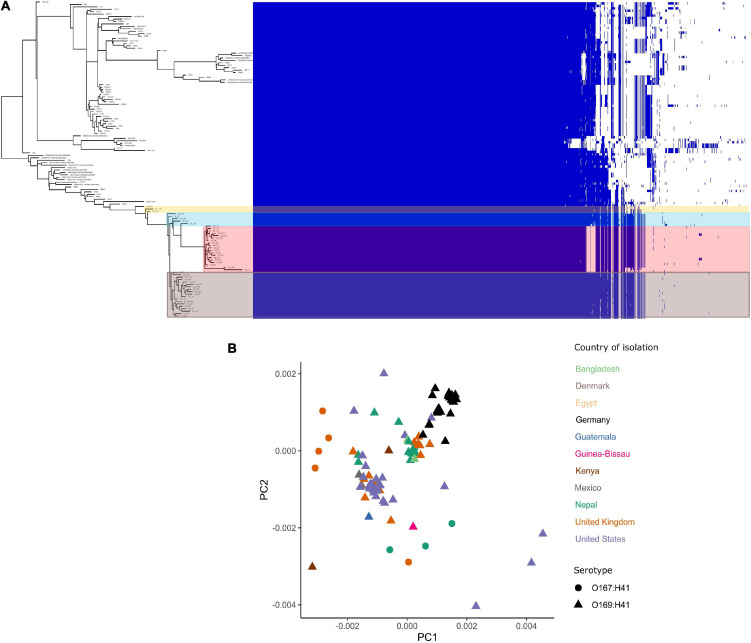
*E. coli* ST182 pangenome. **(A)** Gene presence/absence matrix clustered by accessory genome visualised using Phandango. Airplane waste isolate clusters highlighted. **(B)** MDS illustrating pairwise genome distances calculated using Mash. Isolates colored by country of isolation. All originating from Germany (black) pertain to this collection. Shapes distinguish serotypes.

The pangenome for the 130 *E. coli* ST182 genomes used in the phylogenetic analysis consists of 6339 genes made up of a core genome of 4053 genes (64%) and an accessory genome of 2286 genes. The accessory genome was further broken down into a soft-core genome of 162 genes (present in 95–99% of genomes), a shell genome of 942 genes (15–94%) and a cloud genome of 1182 (<15%). A genome-wide association study identified 1057 genes associated with the airplane waste ST182 lineage, including both genes positively and negatively correlated (Supplementary Data 4). Despite having conserved core genomes, the airplane waste isolates formed 4 clusters of their own based on accessory genome content (Figure 2A). A pairwise genome distance MDS plot (Figure 2B; airplane waste isolates in black) shows that most of the airplane waste isolates formed a clonal group, however, several airplane waste ST182 isolates were in closer proximity to isolates originating from United Kingdom and one isolate from the United States (2014EL-1345-2). Notably, two other clonal groups were evident consisting of isolates originating from diverse geographical regions, including Bangladesh, Nepal and the United Kingdom in one group, and Egypt, United Kingdom and United States in the other.

### *Escherichia coli* ST182 Antimicrobial Resistance Profile

**FIGURE 3:**
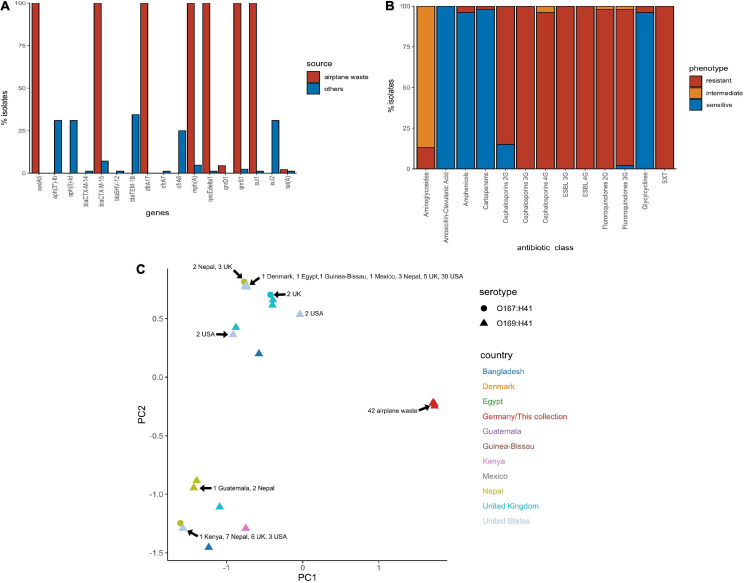
Antimicrobial resistance (AMR) profile of *E. coli* ST182 strains. **(A)** Bar graph comparing distribution of the most common ARGs identified in both ST182 airplane waste isolates (red) and other ST182 strains (blue). **(B)** Phenotypic AMR profile of ST182 airplane waste isolates. Red = resistant isolates (%), orange = intermediate (%), blue = sensitive (%). **(C)** MDS analysis of ARGs detected in ST182 isolates. Where identical profiles cause shapes to overlap arrows have been added detailing the country of isolation for each clustered isolate.

All 130 *E. coli* ST182 genomes were screened for ARGs (Supplementary Data 5). The presence of ARGs typically associated with horizontal gene transfer (HGT) are presented in Figure 3A. Of these HGT-associated ARGs, the airplane waste derived ST182 isolates carried an average of eight while other ST182 strains carried an average of three, and all airplane-derived isolates carried ARGs conferring resistance to ESBLs (*bla*_CTX–M–__15_), streptomycin (*aadA5*), macrolides (*mphA*), fluoroquinolone (*qnrS1*), sulphonamide (*sul1*) and trimethoprim (*dfrA17*) (Figure 3A). The genotypic data was congruent with phenotypic data, and according to the criteria defined by Magiorakos et al. (2012), all *E. coli* ST182 airplane waste isolates were classified as multidrug resistant (Figure 3B and Supplementary Data 6). Conversely, these six specific genes were far less common in other *E. coli* ST182 strains, ranging from 0% for *aadA5* and *dfrA17* to 6% (*n* = 5) for both *bla*_CTX–M–__15_ and *mphA*. Only one other intact gene conferring resistance to ESBLs was identified in other ST182 strains (i.e., *bla*_CTX–M–__14_) identified in one strain (069d2850; Nepal), and only one carbapenem resistance gene (i.e., *bla*_KPC–__3_) also in one strain (2016EL-1001a; United States). The most common ARGs in other ST182 strains were *bla*_TEM–__1__B_ (β-lactam, 34% vs 0% in airplane waste isolates), *aph(3”)-Ib* (aminoglycoside, 33 vs 0%) and *sul2* (sulphonamide, 30 vs 4%) (Figure 3B). An MDS analysis of all ARGs identified using the comprehensive antimicrobial resistance database (CARD), which includes transmissible as well as intrinsic ARGs (such as efflux pumps), demonstrated that overall airplane waste isolates had the most distinct and divergent ARG profiles (Figure 3C; red triangles).

Chromosomal point mutations conferring AMR were also screened for in airplane-derived isolates and all were observed having the S83L mutation (TCG→TTG) in DNA gyrase subunit A gene *gyrA* conveying resistance to nalidixic acid and ciprofloxacin (fluoroquinolone). The same mutation was also found in 25 (30%) other ST182 isolates. No other AMR-associated mutations were identified (Supplementary Data 5). Interestingly, while airplane waste ST182 isolates were resistant to ciprofloxacin, most were less resistant to norfloxacin, including seven isolates that were sensitive (Supplementary Data 6).

Class 1 integrase (*intI1*), as well as transposons and insertion sequences (ISs) associated with mobilising AMR regions were also screened for Supplementary Data 5. Notably, all airplane-derived isolates had at least partial IS*26* and/or the 3 SNP variant IS*15DI* (32 intact [70%], 13 incomplete [30%]), while less than half (45%) of other ST182 strains possessed IS*26*, and none had IS*15DI*. Similarly, while all airplane-derived isolates carried at least a partial *intI1* gene, this gene was only present in three other ST182 strains (3%, 2 intact, 1 partial).

### *Escherichia coli* ST182 AMR Regions

**FIGURE 4:**
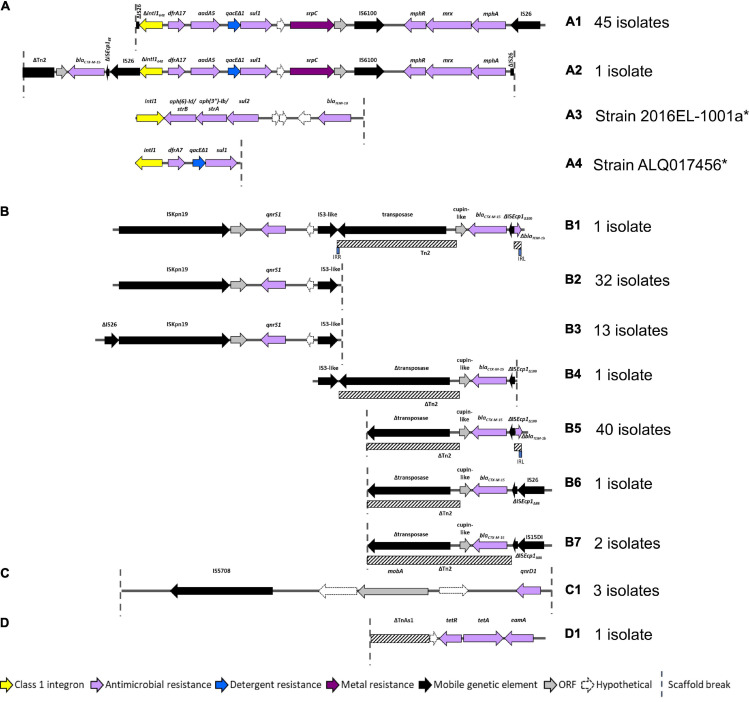
AMR regions in ST182 isolates. **(A)** Structures containing class 1 integrons. *Two structures not from this collection but present in two other ST182 strains analysed in this study. **(B)** Structures containing *bla*_CTX–M–__15_ and/or *qnrS1.* **(C)** Structure carrying *qnrD1*. **(D)** Structure containing *tetA.*

As AMR regions are often abundant in repetitive sequences, assembling complete structures using short-read sequencing data can be challenging. Despite this limitation we resolved four main structures with several variants (Figure 4). BLASTn and NCBI were used to determine whether these AMR regions had been previously deposited into public databases (Table 2).

**TABLE 2:** Summary of BLAST hits (100%) to AMR regions found in this collection.

IS*26* flanked class 1 integron, chromate resistance and macrolide inactivation structure (Figure 4A1)								
Species	Strain	ST	Plasmid name	Accession	pMLST	Host	Location	Date
*E. coli*	118UI	69	pEco118UIb	NZ_CP032516.1	IncF NT	Human	Mexico	2009
*E. coli*	CA08	131	pCA08	CP009233.1	F2:A-:B20	Human	United States	NA
*E. coli*	Ecol_867	131	pEC_867_1	CP018982.1	F-:A-:B20	Human	Canada	2013
*E. coli*	AR_0104	131	unitig_5	NZ_CP020117.1	F1:A-:B-	NA	NA	NA
*E. coli*	DA33135	131	pDA33135-139	NZ_CP029577.1	F29:A-:B10	Human	Sweden	NA
*E. coli*	E41-1	131	p1	NZ_CP028484.1	F1:A-:B20	Human	China	2017
*E. coli*	TO60	131	p2	NZ_LS992169.1	F1:A-:B20	NA	NA	NA
*E. coli*	U15A	131	pU15A_A	NZ_CP035721.1	IncI1-Iγ	Human	United States	2015
*E. coli*	MO	131	pMO	MG886288.1	F1:A-:B20	Water	NA	NA
*E. coli*	AR_0086	131	pUnnamed1	NZ_CP032202.1	F2:A-:B20	NA	NA	NA
*E. coli*	131	131	p146-1	NZ_CP041573.1	IncF NT	Human	United States	NA
*E. coli*	E303	131	pE303_IMP6	AP022363.1	IncF NT	Human	Japan	2015
*E. coli*	425	131	p425	MK295830.1	F1:A6:B20	Human	Israel	2007
*E. coli*	HOS15	131	-	SRR11495735	-	Human	Australia	2006
*E. coli*	HOS58	131	-	SRR11495760	-	Human	Australia	2007
*E. coli*	A1_181	410	p_unnamed3	NZ_CP040070.1	F1:A-:B-	Seagull	United States	2016
*E. coli*	ECONIH1	648	pECO-824	NZ_CP009860.1	F1:A-:B1	Human	United States	2013
*E. coli*	MS14385	648	p2	NZ_LR130556.1	F1:A-:B-	Human	Australia	NA
*K. michiganensis*	K518	NA	Chromosome	NZ_CP023185.1	Chromosome	Human	China	2017
*K. pneumoniae*	F93-2	17	pF93-2_1	NZ_CP026158.1	F2:A-:B-	Human	China	2014
*K. pneumoniae*	FDAARGOS_439	147	pUnnamed	NZ_CP023918.1	F2:A22:B-	Human	Canada	2014
***bla*_CTX–M–__15_ and *qnrS1*-containing structure (Figure 4B1)**								
**Species**	**Strain**	**ST**	**Plasmid name**	**Accession**	**pMLST**	**Host**	**Location**	**Date**
*E. coli*	MRSN346647	38	pMRSN346647_113.1	NZ_CP018207.1	F-:A-:B53	Human	United States	2016
*E. coli*	2014EL-1345-2	182	pUnnamed 3	CP024226.1	IncB/O/K/Z	Human	United States	2014
***tetA*-containing structure (Figure 4D1)**								
**Species**	**Strain**	**ST**	**Plasmid name**	**Accession**	**pMLST**	**Host**	**Location**	**Date**
*E. coli*	ECCWS199	48	pTB222	NZ_CP032239.1	IncN	Chicken	China	2017
*K. pneumoniae*	TH164	873	pTH164-1	NZ_CP035212.1	IncR	Human	Thailand	2012
*S. enterica*	SCSM4.1	34	plas4.1.1	NZ_CP047116.1	IncHI2	Chicken	China	2019
*S. enterica*	sg_wt7	36	Chromosome	NZ_CP036168.1	Chromosome	Food	Singapore	2016

All 46 isolates contained an IS*26* flanked AMR region comprised of a class 1 integron cassette, chromate resistance gene *srpC* (*chrA* homologue), and macrolide inactivation gene cluster *mphA-mrx-mphR* (Figures 4A1,A2). The class 1 integron structure consisted of an IS*26*-mediated *intI1* truncation (Δ*intI1*_648_), *dfrA17, aadA5, qacE*Δ*1* (quaternary ammonium compounds resistance), and *sul1*. The class 1 integron structure present in airplane isolates differed from those identified in other *E. coli* ST182 strains (*n* = 2, one strain each) (Figures 4A3,A4). One isolate (SS_2_D8) also carried *bla*_CTX–M–__15_ upstream to the class 1 integron (Figure 4A2). While the ARG configuration described in Figure 4A2 appears novel (no 100% BLASTn hits), the Figure 4A1 structure found in all airplane isolates also features in three *Klebsiella* spp. strains, and 18 *E. coli* strains (Table 1). Of the *E. coli* strains, all were from pandemic ExPEC lineages (ST69, ST131, ST410, ST648) isolated mainly from human hosts from various geographical locations. The structure was most frequently located on IncF plasmids of varying pMLSTs, but also chromosomally in one *Klebsiella michiganensis* strain (Table 2).

Unlike SS_2_D8, most isolates (*n* = 44) carried *bla*_CTX–M–__15_ on contigs without a downstream integron, and a scaffold break truncating the Tn*2* transposon harbouring this gene (Figures 4B4–7). The Tn*2* transposon typically carries *bla*_TEM–__1__B_ and a 169 bp fragment of *bla*_TEM–__1__B_ was identified (Figures 4B1,B5), but the fragment is removed by IS*26*/IS*15DI*-mediated Tn*2* truncations seen in Figures 4B6,B7. The *bla*_TEM–__1__B_ fragment is immediately adjacent to a IS*Ecp1* fragment, which is either IS*Ecp1*_Δ__88_ in structures 4B6 and B7 (with IS*26*/IS*15D1*) or IS*Ecp1*Δ_100_ in the absence of adjacent IS elements. In one isolate, SS_2_C11, a continuation of the contig upstream revealed an additional AMR region containing fluoroquinolone resistance gene *qnrS1* (Figure 4B1). Given that this region, spanning 5684 bp from IS*Kpn* to an IS*3*-like element, is identical to the *qnrS1-*containing contigs in all other isolates (Figures 4B2,B3), suggests that the structure presented in Figure 4B1 may be common to all isolates, though future long-read sequencing is needed for confirmation. This SS_2_C11 *bla*_CTX–M–__15_ and *qnrS1-* containing structure (Figure 4B1) has only been observed in two publicly deposited genomes; one *E. coli* ST38 strain isolated from a human in the United States (NZ_CP018207.1) and carried on an IncF plasmid, and the other in an IncB/O/K/Z plasmid from ST182 strain 2014EL-1345-2 (closest relative to this collection, Figure 1A).

In addition to *qnrS1*, three isolates, SS_2_B2, SS_2_E8 and SS_2_D8, also carried another fluoroquinolone resistance gene – *qnrD1*. In each instance, this gene was located on the structure described in Figure 4C1 and appears unique to this collection. Only one isolate, SS_2_E8, carried an intact tetracycline gene *tetA*, the genetic context of which is described in Figure 4D1.

### *Escherichia coli* ST182 Virulence Profile

**FIGURE 5:**
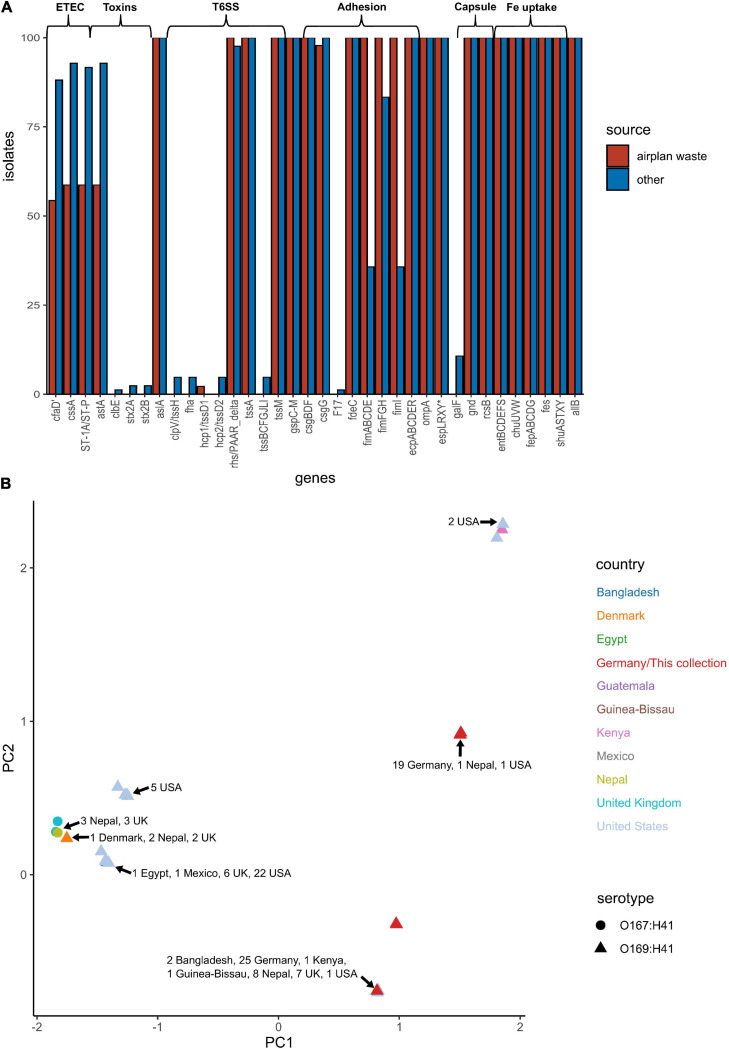
Virulence-associated gene (VAG) profile of *E. coli* ST182 strains. **(A)** Bar graph comparing distribution of VAGs identified in both airplane waste ST182 isolates (red) and other ST182 strains (blue). **(B)** MDS analysis of VAGs detected in ST182 isolates. Where identical profiles cause shapes to overlap, arrows have been added detailing the country of isolation for each clustered isolate.

*E. coli* ST182 genomes were screened for VAGs using ABRicate in conjunction with the VFDB database (Supplementary Data 5). ETEC are defined by the ability to produce heat-liable toxin (LT) and/or heat-stable toxin (ST, subtypes STh and STp), and are characterised by antigenically distinct colonisation factors (CF) and coli surface antigens (CS). The only defining ETEC virulence combination identified in any *E. coli* ST182 was CS6 (*cssA*) + STp, present in 59 and 93% of airplane waste isolates and other ST182 strains, respectively. Similarly, the EAST1 toxin, (*astA)*, was also less prevalent in the airplane waste collection (59%) compared to other ST182 strains (93%). Notably, while the type 1 pili operon was present in all airplane waste isolates, *fimABCDEI* were identified in only 36% of other ST182 stains, though *fimFGH* counts were higher at 83% (Figure 5A). An MDS analysis of VAGs showed that 25 airplane waste isolates shared identical virulence profiles to 20 ST182 strains isolated in Bangladesh, Kenya, Guinea-Bissau, Nepal, the United Kingdom and the United States, and that 19 isolates shared identical virulence profiles to two other ST182 strains, one isolated in Nepal, the other in the United States. The remaining 2 airplane waste isolates had unique virulence profiles (Figure 5B).

**FIGURE 6:**
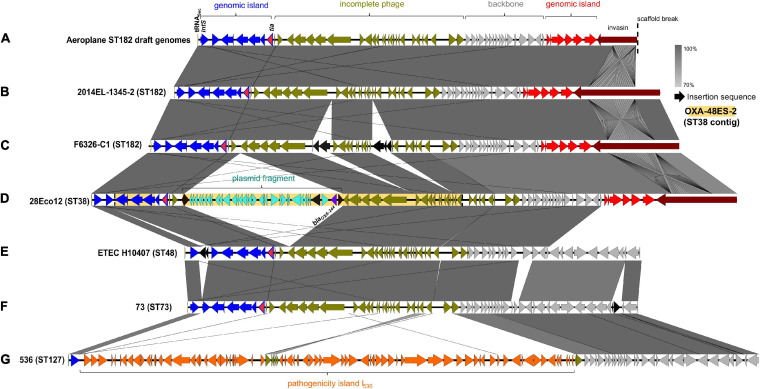
Invasin-enriched region containing *tia*. **(A)** Region represented in isolates from this collection. **(B)** Region located *E. coli* ST182 strain 2014EL-1345-2 (CP024223.1, location: 2157800 – 2225307). **(C)** Region located in *E. coli* ST182 strain F6326-C1 (CP024263.1, location: 3353753 – 3426635). **(D)** Region in *E. coli* ST38 strain 28Eco12 shown in reversed orientation (CP038505.1, location: 48178 – 136524). Shaded yellow region represents genes present a sequenced *E. coli* ST38 contig (KT444705.1). The full contig is represented, which lacks four genes present in 28Eco12, including *tia*, and has no additional genes. **(E)** Region in *E. coli* ST48 (CC10) strain ETEC H10407 (FN69414.1, location: 4215539 – 4277567). **(F)** Region in *E. coli* ST73 strain 73 shown in reverse (CP041538.1, location: 1778685 – 1839792). **(G)** Region in *E. coli* ST127 strain 536 (CP000247.1, location 3947789 – 4041729). Image created using EasyFig.

In addition to the *fim* operon, airplane waste isolates also had a greater occurrence of adhesin/invasin *tia* (89 vs 23%). This virulence factor was found situated on a predicted GI of 10,010 bp, attached at tRNA_*Sec.*_ The GI contained a prophage integrase (*intS*) with 70% amino acid sequence identity to the Enterobacteria phage phiR73 integrase, phosphoethanolamine transferase (*psiE*), GTPase era, and four hypothetical protein encoding genes. The GI was adjacent to a 26,477 phage-like region (predicted to be an incomplete phage by PHAST) and 11,351 bp downstream from the phage-like region was another predicted GI of 6,962 bp. This second GI contained VAGs including invasin *ipaB* and associated chaperone *sicA*, cell invasion protein gene *sipD* and virulence transcriptional regulator *hilA* and was boarded by a gene encoding a protein with invasin, intimin and inverse autotransporter conserved domains. This entire 60,824 bp region (Figure 6A) was present at 100% identity and 99.89–100% coverage in all airplane isolates that carried the *tia* gene, and in other *tia*-carrying ST182 strains at 99.69–100% identity and 80.5–100% coverage (mean coverage of 95.62%) (BLAST results Supplementary Data 7). The region in airline waste isolates shared high nucleotide sequence homology to the same region situated in the complete chromosomes of *E. coli* ST182 strains 2014EL-1345-2 (Figure 6B) and F6326-C1 (Figure 6C) with the main variations occurring due to a scaffold break in our isolates resulting in a truncated boarder invasion/intimin/autotransporter gene (∼5,300 bp vs ∼12,000 bp), and IS*So4*-like (IS*21* family) and IS*679*-like (IS*66* family) insertions within the phage-like region in *E. coli* strain F6326-C1 (Figure 6C). The region was also located in *E. coli* strains ETEC H10407 (ST48) and 73 (ST73), however, these strains lacked the second GI containing *ipaB* and *sipD*, and the large invasion/intimin/autotransporter gene (Figures 6E,F).

Interestingly, this chromosomal region appears to be a hotspot for various insertions, including a typically IncL/M plasmid-associated 20,327 bp Tn*6237* flanked by two IS*1R* elements (Beyrouthy et al., 2014), containing carbapenem-resistance gene *bla*_OXA–__244_ (1 SNP variant of *bla*_OXA–__48_), found in two *E. coli* ST38 strains (Figure 6D) and the uropathogenic *E. coli* (UPEC)-associated pathogenicity island (PAI) PAI I_536_ (Figure 6G).

### Plasmid Replicon Profile

**FIGURE 7:**
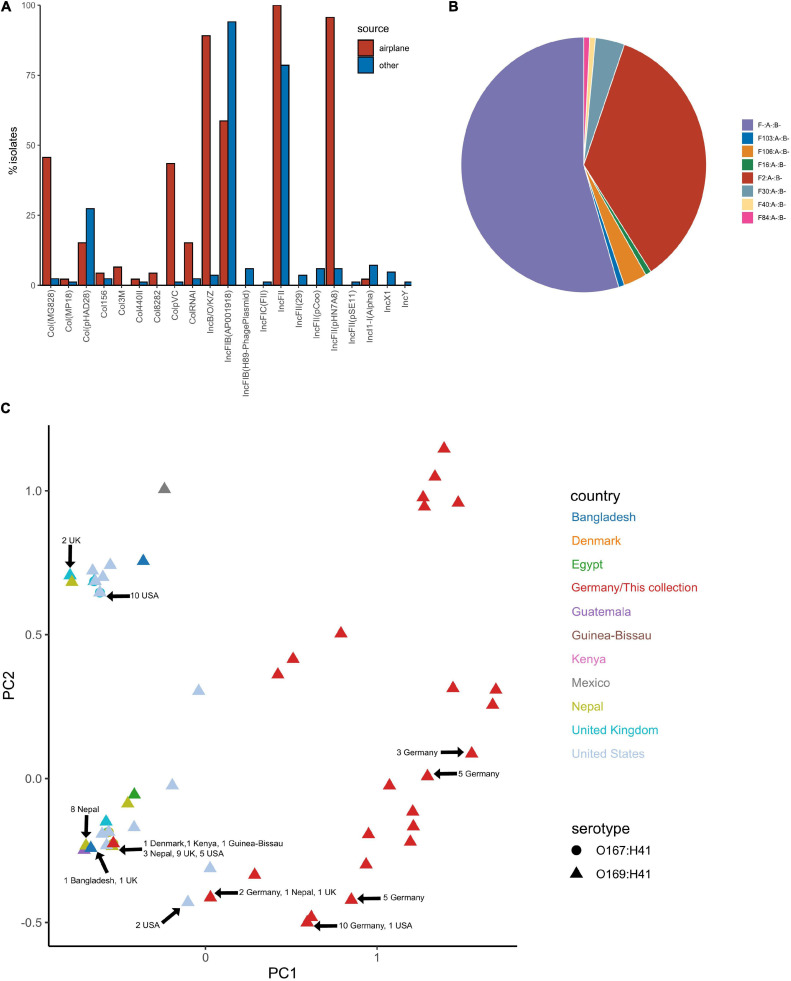
Plasmid replicon profile of *E. coli* ST182 isolates. **(A)** Bar graph comparing the distribution of plasmid replicons identified in airplane waste isolates (red) and other ST182 strains (blue). **(B)** Pie chart illustrating the distribution of F plasmid pMLST. F2:A-:B- (red) is unique to airplane waste isolates. **(C)** MDS analysis of plasmid replicons. Isolates from this collection depicted as red triangles. Where identical profiles caused overlaps, arrows have been added detailing country of origin for each clustered isolate.

Plasmid replicons were screened for using ABRicate in conjunction with the PlasmidFinder database. The plasmid replicon profile for isolates in this collection varied considerably in terms of incompatibility (Inc) types, prevalence and combinations compared to other ST182 strains analysed, as well as within the collection (Figures 7A,C). The number of replicons per airplane waste isolate ranged from 2 to 10, with an average of 5, while in other *E. coli* ST182 strains the number ranged from 0 to 6, with an average of 3 replicons. The most common replicon in this collection was IncFII (AYA58016) (*n* = 46, 100%; vs *n* = 66, 79% of other ST182 strains), followed by IncFII_pHN7A8 (JN232517) (*n* = 44, 96%; vs *n* = 5, 6%) and IncB/O/K/Z (FN868832) (*n* = 41, 89%; vs *n* = 2, 2%). Conversely, the most common replicon in other ST182 strains was IncFIB (AP001918) (*n* = 79, 94%; vs *n* = 27, 59% in airplane isolates), followed by the previously mentioned IncFII (AYA58016, present in all airplane isolates) and Col_pHAD28 (*n* = 23, 27%; vs *n* = 7, 15%). For F plasmids, pMLST identified that all airplane waste isolates carried F2:A-:B- plasmids, and that this pMLST was unique to this group, while F-:A-:B- was the most common pMLST in other ST182 strains (*n* = 71, 85%) (Figure 7B).

**FIGURE 8:**
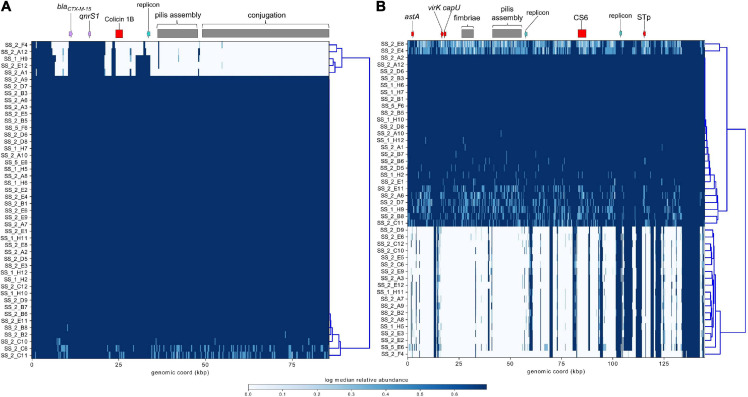
Mapping of short-reads indicating the presence of IncB/O/K/Z and IncF plasmids. **(A)** Schematic and heatmap for IncB/O/K/Z plasmid from *E. coli* ST182 strain 2014EL-1345-2. **(B)** Schematic and heatmap for IncF plasmid from *E. coli* strain 2014EL-1345-2. Clustering of rows is based on the similarity between plasmid coverage profiles. Solid blue color indicates a read depth of over 20, and a light blue color indicates a read depth between 10 and 20.

ST182 strain 2014EL-1345-2 is currently the closest relative to the airplane waste isolates and likewise carries *bla*_CTX–M–__15_. This gene is harboured on an IncB/O/K plasmid (NZ_CP024226.1, 85,864 bp), and was used here as a reference sequence for short read mapping to indicate the presence of a similar plasmid in the airplane waste isolates (Figure 8A). While the AMR region, consisting of *bla*_CTX–M–__15_ and *qnrS1*, was present in all airplane waste isolates, five isolates lacked most of the plasmid contents, including the IncB/O/K replicon in two isolates suggesting that *bla*_CTX–M–__15_ and *qnrS1* are carried on a different Inc type plasmid. Plasmid read mapping was also performed on 2014EL-1345-2 plasmid CP024227.1 (IncF plasmid), which harbours the ETEC-specific VAGs STp and CS6 and shares 99.88% nucleic acid sequence identity with virulence plasmid pEntYN10 (AP014654.2) isolated from an O169:H41 strain that caused an outbreak in Japan (Ban et al., 2015) (plasmid comparisons in Supplementary Data 8). Consistent with VAG screening, 24 (52%) of airplane waste isolates appear to carry a similar plasmid, though lower read-depths in some isolates suggests allelic variations (Figure 8B). Furthermore, the pMLST for the 2014EL-1345-2 plasmid is F-:A-:B-, not F2:A-:B- as carried by airplane waste isolates. Future long-read sequencing studies are needed to provide both greater insight into these initial observations, as well as to investigate plasmids harbouring replicons absent in strain 2014EL-1345-2.

## Discussion

ETEC strains are the leading cause of diarrhoeal disease in humans visiting or residing in developing countries and cause foodborne outbreaks in developed countries. ETEC O169:H41 strains have caused at least 29 outbreaks of gastroenteritis in developed countries, yet to date, no comparative genomic analyses nor reports on AMR pertaining to this ETEC serotype have been published. Here we present 46 draft genomes of O169:H41 (ST182) *E. coli* isolates originating from sewage taken from aircraft landing at an international airport in Germany on a single day in 2016. By comparing these isolates to 84 publicly available ST182 genomes, several key observations were made including: (i) ST182 ETEC carry heat-stable toxin STp and colonisation factor CS6; (ii) the airplane ST182 isolates have conserved core genomes but carry diverse sets of plasmid replicons; (iii) the airplane ST182 lineage is ESBL-producing and fluoroquinolone resistant; and (iv) the airplane waste isolates carry a class 1 integron-containing structure seen in major ExPEC STs such as ST131.

### *Escherichia coli* ST182 Carry Heat-Stable Toxin STp, Colonisation Factor CS6, and a Putative Tia-Associated Genomic Island

Defining features of human ETEC include the presence of plasmid-associated heat-labile enterotoxins (LT) and/or heat-stable enterotoxins (STa) and CFs. These virulence factors are typically carried on IncFIB and IncFII plasmids and occur in various combinations (Johnson and Nolan, 2009). ETEC expressing STa have been described as more virulent than ETEC expressing only LT (Qadri et al., 2005). STa includes variants STh and STp, the latter being first described in porcine ETEC but has since been identified in human and bovine ETEC (Qadri et al., 2005; Dubreuil et al., 2016; Wang et al., 2019). Here we found that the only combination of classical ETEC virulence factors in *E. coli* ST182 was STp + CS6. This virulence combination has been reported as most prevalent in patients with diarrhoea in Thailand (Puiprom et al., 2010), and associated with moderate-severe diarrhoea in traveller’s to Mexico and Guatemala (Nicklasson et al., 2010).

A complete genome of ST182 strain 2014EL-1345-2, isolated in 2014 in the United States (Smith et al., 2018), carrying an IncF plasmid containing both STp and CS6, enabled short-read plasmid mapping to be performed and suggested that approximately half of the airplane-derived ST182 isolates carried similar plasmid cargo. This was congruent with the observation that only 52% of airplane-derived ST182 isolates carried STp + CS6. However, it is important to note that ETEC virulence plasmids have demonstrated instability under laboratory conditions (Crossman et al., 2010; Hazen et al., 2019). Indeed, the pEntYN10 plasmid from a Japanese O169:H41 outbreak strain, sharing high sequence homology to the mapped 2014EL-1345-2 virulence plasmid (99.88%), is unstable *in vitro* (Ban et al., 2015) and other STp + CS6 carrying plasmids have been lost after one overnight passage (Tobias et al., 2016). Therefore, it is possible that the virulence plasmid in some airplane-derived ST182 isolates was lost prior to sequencing.

In addition to enterotoxins and CFs, ETEC are known to carry a number of non-classical virulence factors such as serine protease autotransporter EatA (Patel et al., 2004), adhesins EtpA (Roy et al., 2008), TibA (Lindenthal and Elsinghorst, 2001) and Tia (Mammarappallil and Elsinghorst, 2000), dynamin LeoA (Michie et al., 2014), and enteroaggregative *E. coli* heat-stable enterotoxin EAST1 (Yamamoto and Echeverria, 1996). In agreement with other studies screening non-classical VAGs in ETEC, EAST1 (encoded by *astA*) was the most common in *E. coli* ST182 outside the airplane waste collection (92%), though less common within the collection (59%) (Rivera et al., 2013). However, only one non-airplane waste isolate carried *eatA*, and no *E. coli* ST182 strain or isolate carried genes encoding TibA, EtpA or LeoA. This is consistent with a previous study reporting that TibA and EtpA are rare in STa + CS6 ETEC (Hazen et al., 2019). Nevertheless, we found that *tia* was prevalent in airplane ST182 isolates (89 vs. 23% in other ST182 strains). In addition to a role in adhesion, Tia has been demonstrated to facilitate invasion of intestinal cells (Fleckenstein et al., 1996). The *tia* gene is often situated on a subtilase-encoding pathogenicity island (SE-PAI) (Bondì et al., 2017; Wyrsch et al., 2020), however, in all *tia*-positive ST182 strains and isolates we found the gene on a putative genomic island (GI) attached to tRNA_*SEC*_. Our comparative analysis of this GI and the surrounding region confirmed a previous report that the tRNA_*SEC*_ appears to be a hotspot for various insertions, including AMR plasmid fragments and the entire ExPEC-associated PAI I_536_ (Abril et al., 2019). This locus is significant in the evolution of ETEC.

### *Escherichia coli* ST182 Diversity

Of the 130 *E. coli* ST182 genomes analysed in this study, the majority (93%) were of serotype O169:H41, though a small portion were O167:H41 (7%). Interestingly, the O167 serogroup is associated with both ETEC and enteroinvasive *E. coli* (EIEC) and related to a *Shigella boydii* O-antigen (Gross et al., 1983). All *E. coli* ST182 strains were typed as phylogroup E, which is uncommon, as most ETEC are of phylogroup A and B1 (von Mentzer et al., 2014; Mosquito et al., 2015; Sahl et al., 2017). Indeed, a phylogenetic analysis of 362 ETEC isolates found that isolates of serogroup O169 containing STp + CS6 formed their own clade, L7, and were the only ETEC lineage associated with phylogroup E (von Mentzer et al., 2014).

Our pangenome analysis demonstrated that ST182 genomes share a core genome of 4053 genes and have an accessory genome of 2286 genes. Despite being clonal in nature, the airplane waste isolates could be discerned by their accessory genomes and carried diverse sets of plasmid replicons. ETEC strains have been reported carrying up to six plasmids (Rasko et al., 2008; Crossman et al., 2010) and up to eight plasmid replicons (Hazen et al., 2019). Here we found that airplane ST182 isolates carried two to 10 plasmid replicons with an average of five per isolate. This was significantly higher than other ST182 strains which carried zero to six replicons with an average of three. The types of replicons also differed, with the most prevalent in airplane isolates being IncFII and IncB/O/K/Z versus IncFIB in other ST182, and indeed other ETEC (Hazen et al., 2019). pMLST indicated that all airplane waste isolates carried a F2:A-:B- plasmid, which was not present in any other ST182 strain. F2:A-:B- plasmids have been reported carrying various *bla*_CTX–M_ genes and other ARGs in both humans and animals (Deng et al., 2011; Dahmen et al., 2013; Grami et al., 2014; Díaz-Jiménez et al., 2020).

### *Escherichia coli* ST182 Found in Airplane Waste Is ESBL-Producing

A standout feature of this *E. coli* ST182 lineage was its AMR profile. All airplane-derived isolates were resistant to 3rd and 4th generation extended-spectrum β-lactams, and all carried ESBL gene *bla*_CTX–M–__15_. Conversely, *bla*_CTX–M–__15_ was only identified in six other ST182 strains (7%) and *bla*_CTX–M–__14_ in one other ST182 strain. The prevalence of ESBL-producing ETEC is growing, particularly in association with traveller’s diarrhoea. A study investigating antimicrobial susceptibilities in traveller’s diarrhoea isolates from 2006 to 2008 found ESBL-producing ETEC in 6% of isolates originating from India (Ouyang-Latimer et al., 2011), while in 2011–2017, another study found 43% of traveller’s diarrhoea isolates from visitors to India and Southeast Asia were ESBL-producing ETEC (Guiral et al., 2019). In the latter study, ESBL-production was attributed to *bla*_CTX–M–__15_ and *bla*_CTX–M–__27_ carriage. Similarly, a study surveying clinical ETEC in Nepal from 2001 to 2016 found only one ESBL-producing ETEC isolate between 2001 and 2009 while over 30% of ETEC isolates collected post 2013 were ESBL-producing, 80% of which carried *bla*_CTX–M–__15_ (Margulieux et al., 2018). These significant increases in ESBL-producing ETEC from the early-mid 2000s to the following decade coincides with the period of rapid global expansion of pandemic ESBL-producing ExPEC lineages, most notably ST131 H30Rx (Zilberberg and Shorr, 2013; Poolman and Wacker, 2016). Additionally, ExPEC have recently been identified as the most common pathotype associated with travel-acquired ESBL-producing *E. coli* (Kantele et al., 2020). Given that ExPEC can colonise the gastrointestinal tract (Manges and Johnson, 2015) and that *bla*_CTX–M–__15_ and *bla*_CTX–M–__14_ (both strongly associated with ExPEC) are most frequently carried on IncF plasmids (Bevan et al., 2017), it is possible that ExPEC have contributed to the evolution of ESBL-production in ETEC (or vice versa) via horizontal gene transfer within a shared environment. However, in ExPEC the majority of *bla*_CTX–M_ genes are associated with IS*Ecp1* (also known as IS*Ec9*), which can capture and mobilise *bla*_CTX–M_ genes and other ARGs (Carattoli, 2013, 2009; Li et al., 2015), but in the airplane-derived ST182 lineage we found that only a 88 bp or 100 bp fragment of IS*Ecp1* adjacent to the *bla*_CTX–M–__15_ genes. In each isolate, *bla*_CTX–M–__15_ was found situated within a Tn*2* transposon and adjacent to a cupin-like gene. Downstream to *bla*_CTX–M–__15_ we identified a 169 bp fragment of *bla*_TEM–__1__B_, the passenger gene of Tn*2* (Siguier et al., 2006; Bailey et al., 2011). This *bla*_CTX–M–__15_ context, including the *bla*_TEM–__1__B_ fragment, has only been described once in the literature and was present in ETEC O159:H20 strains responsible for the first ESBL-associated foodborne outbreak in South Korea in 2016 (Kim et al., 2017).

### *Escherichia coli* ST182 Found in Airplane Waste Is Fluoroquinolone Resistant

Like ESBL-production, fluoroquinolone resistance in ETEC strains has also been increasing, particularly in isolates originating from traveller’s visiting India, Southeast Asia and Africa (Mendez Arancibia et al., 2009; Guiral et al., 2019). Between 2001 to 2007, it was reported that 8% of ETEC strains from India and Southeast Asia were resistant to fluoroquinolones (Mendez Arancibia et al., 2009), increasing to 43% in 2011–2017 (Guiral et al., 2019). In this study, all airplane-derived ST182 isolates were phenotypically resistant to fluoroquinolones and all carried the plasmid-associated fluoroquinolone resistance gene *qnrS1*, with three isolates also carrying *qnrD1.* The plasmid-associated *qnrD1* gene is relatively uncommon and generally only reported in Proteeae (*Proteus* spp., *Morganella morganii* and *Providencia stuartii*); however, a recent study on 183 *Enterobacteriaceae* sourced from health care facilities in India found 23.5% harboured *qnrD1* indicating dissemination to other species including *E. coli* and *Klebsiella* spp. (Dasgupta et al., 2017). Only two ST182 strains outside this collection (2%) carried *qnrS1*, and no other fluoroquinolone resistant genes were identified. One *qnrS1*-carrying ST182 strain was again 2014EL-1345-2, the closest relative to this collection. The *qnrS1* gene in 2014EL-1345-2 is situated on a resolved IncB/O/K/Z plasmid, along with *bla*_CTX–M–__15_ upstream to *qnrS1*. Short-read plasmid-mapping exercises suggested that most airplane-derived isolates carried a similar plasmid. However, future long-read sequencing is required to confirm this observation as mapped reads are taken from the entire genome and do not confirm genetic context.

In addition to plasmid-associated fluoroquinolone genes, we identified a fluoroquinolone-conferring S83L mutation in *gyrA* (Ruiz, 2003) in all airplane isolates and 30% of other ST182 isolates. Mutations conferring resistance to fluoroquinolone in *gyrA* and in DNA topoisomerase 4 subunit A *parC* have been described in ETEC previously (Mendez Arancibia et al., 2009), and specifically the S83L mutation was identified in ETEC outbreak strains of various serotypes in India (Chakraborty et al., 2001; Pazhani et al., 2011).

### Airplane Waste *Escherichia coli* ST182 Carry a Class 1 Integron Found in ExPEC

Class 1 integrons are considered a reliable proxy for a multidrug resistant genotype and have played an important role in the spread of AMR in *Enterobacteriaceae* (Gillings, 2014). Class 1 integrons are often associated with mobile genetic elements, such as plasmids and IS elements, particularly IS*26* (Partridge et al., 2018), which enable lateral dissemination of ARG cargo. Here we found that, while class 1 integrons were rare in other ST182 (3%) strains, all airplane waste ST182 isolates carried a class 1 integron upstream of a macrolide inactivation gene cluster (*mphR-mrx-mphA)*. This complex resistance region (CRR) was flanked by IS*26* elements and was comprised of IS*26*-Δ*intI1*_648_-*dfrA17-aadA5-qacE*Δ*1-sul1-*ORF-*srpC-padR-IS*6100-*mphR-mrx-mphA-*IS*26*. The *dfrA17-aadA5* integron (In54) cassette was first characterised in 2000 after its discovery in an *E. coli* isolated from a urinary tract infection (UTI) patient in Australia (White et al., 2000). Since then, *dfrA17-aadA5* has become one of the most prevalent cassette arrangements worldwide in *E. coli* (Yu et al., 2004; Xia et al., 2016; Kaushik et al., 2019) and in other *Enterobacteriaceae* including *Salmonella* species (Meng et al., 2017), and is thought to have driven trimethoprim resistance in ExPEC ST69 strains (also known as clonal group A) (Solberg et al., 2006). While the *dfrA17-aadA5* cassette has been previously identified in integrons present in clinical ETEC strains (Kartsev et al., 2015), a BLASTn search demonstrated that the entire *IS*26 flanked CRR containing the integron found in ST182 airplane isolates has not been observed in ETEC previously, but in *Klebsiella, Salmonella* and ExPEC-associated STs, most commonly in ST131. In ST131 strains, this CRR is distributed globally, is carried on various F plasmids, and is harboured by isolates of various origins including humans (Li et al., 2020) and oysters (Fernandes et al., 2020).

In conclusion, within the context of growing reports of rising antimicrobial resistance in travel acquired *Enterobacteriaceae*, we provide the first WGS analysis of antimicrobial and virulence gene carriage in ETEC ST182 isolates originating from airline passengers arriving in Germany from abroad. Our data indicates that these isolates constitute a new lineage of ST182 that is resistant to multiple, clinically important antibiotics. Overall, this study contributes to a better understanding of the spread of ETEC as well as AMR worldwide.

## Data Availability Statement

The datasets presented in this study can be found in online repositories. The names of the repository/repositories and accession number(s) can be found below: https://www.ncbi.nlm.nih.gov/bioproject/639663, SAMN15244685 – SAMN15244730.

## Author Contributions

VJ: conceptualisation, formal analysis, investigation, visualisation, and writing – original draft. SH: data curation and investigation. KA: investigation. TB: funding acquisition and project administration. SD: conceptualisation, funding acquisition, project administration, and supervision. All authors contributed to writing – review and editing.

## Conflict of Interest

The authors declare that the research was conducted in the absence of any commercial or financial relationships that could be construed as a potential conflict of interest.

## Publisher’s Note

All claims expressed in this article are solely those of the authors and do not necessarily represent those of their affiliated organizations, or those of the publisher, the editors and the reviewers. Any product that may be evaluated in this article, or claim that may be made by its manufacturer, is not guaranteed or endorsed by the publisher.
